# Predisplacement Abuse and Postdisplacement Factors Associated With Mental Health Symptoms After Forced Migration Among Rohingya Refugees in Bangladesh

**DOI:** 10.1001/jamanetworkopen.2021.1801

**Published:** 2021-03-16

**Authors:** Ahmed Hossain, Redwan Bin Abdul Baten, Zeeba Zahra Sultana, Taifur Rahman, Mirza Asif Adnan, Moynul Hossain, Taifur Aziz Khan, Muzakkir Kamar Uddin

**Affiliations:** 1 Department of Public Health, North South University, Bashundhara, Dhaka, Bangladesh; 2 Health Management BD Foundation, Cox’s Bazar, Bangladesh; 3 Global Health Institute, North South University, Bangladesh; 4 College of Public Health, University of Iowa, Iowa City; 5 Cambridge Programme to Assist Bangladesh in Lifestyle and Environmental Risk Reduction, University of Cambridge, Cambridge, United Kingdom; 6 International Organization for Migration, Cox’s Bazar, Bangladesh

## Abstract

**Importance:**

At the end of August 2017, violence and persecution in Myanmar’s Rakhine state forced nearly 1 million Rohingyas to flee to Bangladesh for their lives and seek shelter. Many refugees, after their traumatic experiences leaving Myanmar, experience mental health problems.

**Objectives:**

To identify the prevalence of posttraumatic stress symptoms (PTSSs) among displaced Rohingya adults and investigate the association of predisplacement abuse and postdisplacement factors with PTSSs.

**Design, Setting, and Participants:**

This cross-sectional analysis from a household survey of 1184 Rohingya adults aged 18 years or older was conducted in 8 refugee camps within Cox’s Bazar, Bangladesh, from September 17, 2019, to January 11, 2020.

**Main Outcomes and Measures:**

The Impact of Event Scale–Revised was used to assess PTSSs. The possible range of scores was 0 to 88; moderate PTSSs were classified using a score cutoff of 33 to 38 and severe PTSSs were classified using a score cutoff of 39 and above. Adjusted prevalence ratios (aPRs) were estimated using a multivariable logistic regression model adjusted for potential confounders.

**Results:**

Of 1184 participants (625 men [52.8%]; mean [SD] age, 35.1 [13.4] years), 552 (46.6%) had severe PTSSs, and 274 (23.1%) had moderate PTSSs. In Bangladesh, refugees are not legally permitted to work in refugees camps, but 276 of 1165 respondents (23.7%) had temporary paid jobs. Moreover, 113 of the 276 working participants (40.9%) and 430 of the 889 nonworking participants (48.4%) reported severe PTSSs. A total of 496 respondents (41.9%) reported inadequate humanitarian aid for their families, and among them, 281 (56.7%) reported severe PTSSs. A total of 136 of 1177 respondents (11.6%) experienced both physical and sexual abuse in Myanmar, and 87 (64.0%) of them had severe PTSSs. The multivariable analysis showed a reduced risk of PTSSs with appropriate humanitarian assistance (aPR, 0.50; CI, 0.38-0.65). Experiencing both physical and sexual abuse before displacement had a significant association with PTSSs (aPR, 2.09; CI, 1.41-3.07). Opportunities for paid employment in refugee camps also reduced the risks of PTSSs (aPR, 0.69; CI, 0.52-0.91).

**Conclusions and Relevance:**

The high prevalence of self-reported severe PTSSs in Rohingya refugees suggests that the trauma of displacement and the violent consequences of military crackdowns still exist. In the Rohingya camp settings of Bangladesh, employment opportunity and sufficient humanitarian aid hold promise as potential interventions to reduce the high prevalence of severe PTSSs. Mental health symptoms were more prevalent in adults who experienced physical abuse or physical and sexual abuse before displacement.

## Key Points

**Question:**

What is the prevalence of severe mental health symptoms among Rohingya refugees in Bangladesh?

**Findings:**

This cross-sectional study including 1184 Rohingya refugees found that a high prevalence of severe mental health symptoms exists in the Rohingya population even after 2 years of displacement. In Rohingya camp settings, low prevalence of traumatic distress was associated with employment opportunities and adequate humanitarian support; severe mental health symptoms were commonly found in adults who had experienced physical abuse or both physical and sexual abuse in Myanmar.

**Meaning:**

The risk of mental illness may be high for the Rohingya population; health care networks in Rohingya camp settings should ensure adequate mental health services to protect Rohingya adults from developing mental illness.

## Teaser

This cross-sectional study examines the prevalence of posttraumatic stress symptoms among displaced Rohingya adults and investigates the association of predisplacement abuse and postdisplacement factors with posttraumatic stress symptoms.

## Introduction

The Rohingya population of Myanmar is one of the world’s most oppressed minority groups.^1^ The Citizenship Act of 1982 removed the Rohingya from the list of officially recognized ethnic minority groups. It denied them many fundamental rights, including citizenship, freedom of movement, access to health care and education, marital registration, and the ability to vote, making them the world’s largest stateless group.^2^ In August 2017, in continuation of past persecution events, the Myanmar army began a massive clearance operation in Rakhine, which was then home to approxmately 1.2 million Rohingya individuals.^3^ The operation saw an estimated 7800 Rohingya killed.^4^ The movement drove the Rohingya out of Myanmar, leading to a massive exodus of about 750 000 Rohingya refugees to Bangladesh’s Cox’s Bazar district.^5^

Several studies have recorded the experience of abuses faced by Rohingya refugees before displacement.^6,7,8,9,10,11^ Rohingya refugees were refused medical care for physical or sexual assault in Myanmar.^7,8,9,10,11,12^ A major component of these violent incidences was also emotional and verbal abuse. Rohingya group leaders echoed and corroborated these descriptions in different reports.^4,12^ These occurrences of physical, sexual, and emotional abuse were reported to have a lasting effect on the survivors’ mental health and to be associated with the potential diagnosis of psychological conditions, such as posttraumatic stress disorder (PTSD).^7^

Posttraumatic stress is a syndrome of PTSD characterized by distracting ideas, nightmares, memories of past traumatic events, avoidance of trauma reminders, hypervigilance, and sleep disturbance.^13^ Such aspects are associated with extreme psychological, occupational, and interpersonal dysfunction.^14^ However, patients with PTSD experience pronounced cognitive, affective, and behavioral reactions to stimuli, resulting in hallucinations, extreme anxiety, and fleeing or combative behavior. These symptoms may result in emotional numbness and reduced involvement in daily activities and, in the extreme, may result in alienation from others. Among patients with PTSD, depressive disorders, anxiety disorders, and drug misuse are 2 to 4 times more prevalent than among patients without PTSD.^15^ Moreover, PTSD may increase the risk of suicide attempts.^16^ We used the Impact of Event Scale–Revised (IES-R) for the evaluation of posttraumatic stress symptoms (PTSSs).^13,14^ This self-report instrument was designed to include all 3 groups of symptoms of PTSD (ie, interference, avoidance, and hyperarousal) associated with a particular life-threatening incident.

During the predisplacement and postdisplacement periods, refugees faced multiple stressors.^17^ Individuals with an experience of abuse were at risk of increasing mental health problems.^18,19^

The challenges facing Rohingya refugees while living in Bangladesh have been reported in many studies.^20,21^ They live in small, overcrowded temporary shelters in refugee camps without adequate food, clean water, or toilets. Moreover, their lives are on hold, and they are unsure about their future. A 2017 study analyzed the daily environmental stressors among 148 Rohingya adults and found worse mental health outcomes for refugees.^21^ Compared with 2 years ago, the Rohingya refugees’ basic needs and health care have mostly improved.^22^ However, postdisplacement factors are still important for improving the mental health of Rohingya refugees.

Screening is effective only when combined with high-quality services for mental well-being. One of the challenges to ensuring appropriate services for Rohingya refugees in Bangladesh is the lack of statistical data on the group’s mental health status. In this report, we plan to identify the prevalence of PTSSs and associated predisplacement and postdisplacement factors of PTSSs among Rohingya adults living in Bangladesh after the massive clearance operation.

## Methods

### Study Design and Participants

From September 17, 2019, to January 11, 2020, we conducted a cross-sectional survey among Rohingya refugees residing in Cox’s Bazar, Bangladesh. More than 1 million Rohingya refugees are now staying in Bangladesh. Most are clustered in Ukhia and Teknaf, the district’s 2 upazilas (administrative regions). The largest refugee settlement in the world, Kutupalong, centered in Ukhia, is home to more than 600 000 refugees alone.^23^ The prospective participants were recruited from Kutupalong via the process of a 3-stage sampling technique. First, with the assumption of equal population size in each camp, we chose 8 camps randomly from the Kutupalong refugee camp and expansion areas that consist of 23 camps. Second, we selected at least 160 households from each selected camp, with a target of 1280 households in the study. We targeted more households to be included in our study than the required sample size at 80% power, 95% CI of 0.05 to 1.96, with an assumption that 40% of the population had a mental illness, and a design effect of 2. We applied a systematic sampling technique, and the first household was randomly chosen from the approximate geographical center of the camp. Data collectors proceeded to the next closest household until 160 households were sampled. Third, we had a single respondent per household interviewed, preferably the head of the household. The female head of the household or other available adult member of the household was surveyed when the male head of the household was not available. Household members have been described as those who lived for at least 1 month under the same roof and shared cooking and eating facilities from the same source. We also ensured that the participant lived in the camp for at least 2 years after the displacement. The details of the sampling allocation are given in eTable 1 in the Supplement. Participants provided verbal consent for the study because they were reluctant to sign their names or provide their fingerprints on any piece of paper; in addition, most participants were analphabetic. They were reassured that all the information collected would be kept strictly confidential and would not be used for anything other than research purposes. However, they were provided with a consent paper with detailed contact information of the research investigators for any future query. The institutional review board at North South University, Bangladesh, approved the study. This study followed the Strengthening the Reporting of Observational Studies in Epidemiology (STROBE) reporting guideline.

### Recruitment and Training

The data were obtained and cleaned by a team of 4 enumerators consisting of 2 men and 2 women from the Health Management BD (HMBD) Foundation. The HMBD Foundation, a local nongovernmental organization (NGO) that has been working in Rohingya refugee camps since the influx, chose the local community leaders (called a “maji”) from each of the selected 8 camps. The local leader from each of the camps was then informed about the study’s research and ethics. They assisted the HMBD Foundation in recruiting 8 local male residents from each camp who could communicate in both the Bengali (Bangla) and Rohingya languages. A team of data collectors was then formed and included 2 persons (ie, one person from the camp and another person from the HMBD Foundation). The interview was held in both the Rohingya and Bangla languages: the camp data collector asked questions in the Rohingya language, and then the HMBD Foundation member checked the answer by asking the same question in Bangla. Our 2 research investigators from North South University (A.H. and T.A.K.) arranged a 1-day practical training session about ethics and data collection. The enumerators and data collectors were also briefed about the study objectives, methods, and questionnaire. The researchers also taught the data collectors about the techniques of report building and preserving neutrality as well as information on ethical problems, privacy concerns, cultural awareness, and risk management for mental health. After the training, a pilot study was arranged for the 8 study teams and was evaluated as a single unit. The aim was to observe the capacity to comprehend the relevant techniques and troublesome situations while interviewing. We made necessary corrections after the pilot study. Afterward, each trained team visited their designated camp together to collect the data using a semistructured questionnaire.

### Data Collection

It was made clear to the respondents that participation in the study was entirely voluntary. The face-to-face interview took place 1 person at a time, to ensure privacy. The respondents were given no monetary or food-item incentives. The questions were read aloud to the respondents 1 question at a time during the interview, and the respondents were asked which of the scale choices was acceptable. The coinvestigators reviewed the data collection sheets for completeness, accuracy, and internal consistency, which were confirmed by the principal investigator.

### Sociodemographic Variables

The first section of the questionnaire assessed sociodemographic characteristics, including age (in years), sex (male or female), height (in inches), weight (in kilograms), marital status (never married, widowed or divorced, and married), and the number of family members in a household. Later we categorized family members as 2 or less, 3 to 4, and more than 4 members. Educational level was subdivided into 2 groups: respondents who attended school and those who cannot read or write.

### Predisplacement Abuse

We used the word “abuse” here, which involves the violence recognized by the intention of imposing control or dominance over Rohingya people to cause rage, harm, resentment, humiliation, coercion, and helplessness. We collected data on the types of abuse that refugees were exposed to before the forced displacement to Bangladesh. The categories were indirect abuse or not exposed to any abuse, verbal or emotional abuse, physical abuse, and both physical and sexual abuse. Verbal or emotional abuse was defined as a nonphysical act that impairs an individual’s psychological integrity and may take the form of coercion, defamation, verbal abuse, or harassment.^24,25^ It might also include being forced into labor and separation from or witnessing abuse against family or community members. Physical abuse was regarded as any force applied to any part of the body, such as shaking, burning or scalding, choking, hair pulling, hitting, slapping, kicking, or threatening and/or attacking with a knife, gun, or other type of weapon. It also also might have included restraining, tying, or locking up against the individual’s will.^26^ Sexual abuse was defined as sexual humiliation (forced masturbation or nudity), sexual slavery, rape (vaginal, oral, anal, or attempted), genital abuse (beatings, electric shock, or mutilation), castration, penis amputation, sterilization, or forced marriage, cohabitation, or sexual activity (with a stranger, family member, or corpse).^26,27^ However, to differentiate between physical and sexual abuse, the respondents were intentionally asked with caution whether the specific event included the sexual organs or not. Because Rohingya women were reluctant to respond to the question of sexual abuse as an individual option, we added the option of both physical and sexual abuse together. Another response option was that the refugee did not experience any direct abuse but was exposed to grief for losing family members or property or experiencing separation, anxiety, or other trauma.

### Postdisplacement Factors

Many of the Rohingya refugees worked in the camp, especially as day laborers who performed critical infrastructure work. In addition to assisting with outreach and coordination in the sprawling camps, the paid volunteer work was essential to initiatives to build roads, prevent landslides, and clear sewage. We included employment status as either employed (paid work) or as unemployed. Based on an individual’s perception, refugees were also assessed on whether they were receiving sufficient humanitarian aid for the family or not. In addition, we obtained data on the presence of any existing physical disability (eg, deafness, blindness, or amputation) of a stateless refugee.

### Outcome Measurement: PTSSs

The study applied the psychometric properties of the IES-R criteria to assess the severity of PTSSs within the study population. This scale is a short, 22-item self-report questionnaire that is not a diagnostic tool for PTSD but an appropriate instrument to measure the subjective response to a specific traumatic event experienced by an adult.^13,28^ Three clusters of symptoms are included in the IES-R: the hyperarousal, interference, and avoidance subscales. Respondents were asked to describe a particular event and then indicate how much each event identified has upset or disturbed them during the past 7 days. We calculated the total subjective stress IES-R score (range, 0-88) from the 3 subscales. The total scale of Cronbach α was 0.87 (95% CI, 0.86-0.88), indicating a high degree of reliability. Posttraumatic stress symptoms were categorized into 4 categories: no symptoms (total IES-R score, ≤23), mild symptoms (score, 24-32), moderate symptoms (score, 33-38), and severe symptoms (score, ≥39) of PTSD. The categorization and interpretation of the IES-R score were described in a report from the Hartford Institute for Geriatric Nursing.^29^ In addition, we categorized the total IES-R score into 2 groups following the recommendation of Creamer et al^13^: less than 33, which identified refugees with no or minimum symptoms of PTSD, and 33 or above, which identified refugees with moderate or severe symptoms of PTSD.

### Statistical Analysis

Data were analyzed using R, version 3.6.2 (R Project for Statistical Computing). The questionnaire, R scripts, and data are available online.^30^ All categorical variables (presented as frequencies and percentages) were assessed using descriptive statistics. We estimated prevalence ratios (PRs) and their 95% CIs using a multivariable logistic model after adjusting for potential confounders. The PR is the ratio between the likelihood of an outcome in the exposed group and the likelihood of an outcome in the unexposed group.^31^ By using the delta method, we obtained the SEs for PRs. In the adjusted model, we controlled for demographic factors, such as age, sex, educational level, marital status, history of predisplacement abuse, current paid employment status in refugee camps, number of family members, and self-reported sufficient humanitarian aid for the family. We also obtained variance inflation factors in the logistic regression model to evaluate potential multicollinearity in the model (eTable 2 in the Supplement).

The pattern of missing data in the study sample is presented in eTable 3 in the Supplement. We found that the proportion of missing data ranged from 0.01% (1 of 1183) for family size to 12.5% (148 of 1036) for body mass index (BMI; calculated as weight in kilograms divided by height in meters squared). Also, we did not include missing data for covariates for 32 participants in multivariable analysis.

## Results

### Response Rate

Of the 1280 households sampled, 17 were excluded because the head of the household did not consent to participate. An additional 57 households were excluded because no persons were eligible to be included in the study during the study period. Finally, in our analysis, we had 1184 households, for a 92.5% response rate. A thorough calculation of the response rate is given in the eAppendix in the Supplement.

### Characteristics of the Participants

Table 1 shows the sociodemographic, predisplacement, and postdisplacement factors of the participants according to the categories of severity of PTSSs. Of the 1184 Rohingya refugees who participated, 625 (52.8%) were men, and 559 (47.2%) were women. The mean (SD) age of respondents was 35.1 (13.4) years. We found that 509 of 1036 respondents (49.1%) had a normal BMI between 18.5 and 24.9, and 431 (41.6%) were overweight or obese. A total of 994 of 1182 respondents (84.1%) were married, and 766 of 1182 respondents (64.8%) had a family of more than 4 members. In addition, 751 of 1156 respondents (65.0%) did not go to school and could not read or write. In Bangladesh, refugees are not legally permitted to work, but 276 of 1165 respondents (23.7%) reported having paid employment opportunities in the refugee camps. We found that 136 of 1177 respondents (11.6%) experienced predisplacement physical and sexual abuse in Myanmar. Sixty-four of 1183 respondents (5.4%) had physical disabilities. Also, 496 of 1182 respondents (42.0%) reported that they did not receive adequate humanitarian aid for their family during the last 7 days.

**Table 1.** Characteristics of Participants by Severity of Posttraumatic Stress Symptoms

Characteristic	Mental health symptoms, No. (%)				
	None (n = 131)	Mild (n = 227)	Moderate (n = 274)	Severe (n = 552)	Total (N = 1184)
Sex (n = 1184)					
Male	54 (8.6)	123 (19.7)	145 (23.2)	303 (48.5)	625 (52.8)
Female	77 (13.8)	104 (18.6)	129 (23.1)	249 (44.5)	559 (47.2)
Age, y (n = 1184)					
18-24	66 (23.7)	63 (22.6)	47 (16.8)	103 (36.9)	279 (23.6)
25-34	44 (11.3)	88 (22.6)	104 (26.7)	153 (39.3)	389 (32.9)
35-44	13 (6.2)	28 (13.4)	45 (21.5)	123 (58.9)	209 (17.7)
45-54	4 (2.2)	26 (14.4)	43 (23.9)	107 (59.4)	180 (15.2)
≥55	4 (3.1)	22 (17.3)	35 (27.6)	66 (52.0)	127 (10.7)
BMI (n = 1036)					
Normal	62 (12.2)	91 (17.9)	100 (19.6)	256 (50.3)	509 (49.1)
Overweight or obese	39 (9.0)	91 (21.1)	121 (28.1)	180 (41.8)	431 (41.6)
Underweight	12 (12.5)	22 (22.9)	17 (17.7)	45 (46.9)	96 (9.3)
Marital status (n = 1182)					
Never married	33 (21.6)	39 (25.5)	29 (19.0)	52 (34.0)	153 (12.9)
Ever married but currently no partner	2 (5.7)	4 (11.4)	9 (25.7)	20 (57.1)	35 (3.0)
Married	96 (9.7)	183 (18.4)	235 (23.6)	480 (48.3)	994 (84.1)
Family size (n = 1182)					
≤2	9 (14.5)	21 (33.9)	10 (16.1)	22 (35.5)	62 (5.2)
3-4	50 (14.1)	86 (24.3)	70 (19.8)	148 (41.8)	354 (29.9)
≥5	72 (9.4)	120 (15.7)	192 (25.1)	382 (49.9)	766 (64.8)
Educational level (n = 1156)					
Cannot read or write	87 (11.6)	121 (16.1)	180 (24.0)	363 (48.3)	751 (65.0)
1-10 Years of schooling	43 (10.6)	101 (24.9)	84 (20.7)	177 (43.7)	405 (35.0)
Paid employment status in the last month (n = 1165)					
Unemployed	89 (10.0)	169 (19.0)	201 (22.6)	430 (48.4)	889 (76.3)
Employed and have earnings	42 (15.2)	54 (19.6)	67 (24.3)	113 (40.9)	276 (23.7)
Physical disability (n = 1183)					
Yes	2 (3.1)	6 (9.4)	15 (23.4)	41 (64.1)	64 (5.4)
No	129 (11.5)	220 (19.7)	259 (23.1)	511 (45.7)	1119 (94.6)
Humanitarian aid for household during the last 7 d (n = 1182)					
Sufficient	82 (12.0)	182 (26.5)	152 (22.2)	270 (39.4)	686 (58.0)
Not sufficient	49 (9.9)	44 (8.9)	122 (24.6)	281 (56.7)	496 (42.0)
Predisplacement abuse (n = 1177)					
Not directly exposed	40 (13.7)	81 (27.6)	67 (22.9)	105 (35.8)	293 (24.9)
Verbal or emotional	68 (14.8)	69 (15.1)	113 (24.7)	208 (45.4)	458 (38.9)
Physical	21 (7.2)	67 (23.1)	54 (18.6)	148 (51.0)	290 (24.6)
Both physical and sexual	1 (0.07)	8 (5.9)	40 (29.4)	87 (64.0)	136 (11.6)
Lost immediate family member during predisplacement violence (n = 342)					
Yes	1 (2.1)	1 (2.1)	9 (18.8)	37 (77.1)	48 (14.0)
No	32 (10.9)	74 (25.2)	57 (19.4)	131 (44.6)	294 (86.0)

Abbreviation: BMI, body mass index.

Moreover, we investigated the association between postdisplacement factors and demographic variables (eFigure in the Supplement). It appears that Rohingya women were not receiving opportunities for paid employment equal to the opportunities that men received. People younger than 45 years were more engaged in paid employment. Also, adults younger than 35 years reported experiencing more physical and sexual abuse relative to older age groups. The Rohingya adults who had more than 4 family members reported a lack of relief from humanitarian agencies.

### Prevalence of PTSSs and Unadjusted PRs

Of the 1184 adult Rohingya refugees, 552 (46.6%) had severe PTSSs, and 274 had moderate PTSSs (23.1%). Severe mental health symptoms were more prevalent in male refugees (303 of 625 [48.5%]) than in female refugees (249 of 559 [44.5%]) (Table 1). The prevalence of severe PTSSs was 57.4% (296 of 516) for refugees aged 35 years or older and 38.3% (256 of 668) for those younger than 35 years. The pattern of severe PTSSs increased with increasing age of the respondents: 18 to 24 years, 36.9% (103 of 279); 25 to 34 years, 39.3% (153 of 389); 35 to 44 years, 58.9% (123 of 209); 45 to 54 years, 59.4% (107 to 180); and 55 years or older, 52.0% (66 of 127). Among married respondents, 480 of 994 (48.3%) had severe PTSSs, while among the never-married respondents, 52 of 153 (34.0%) had severe PTSSs. Of the 766 households with more than 4 family members, 382 (49.9%) had severe PTSSs; of the 62 households with less than 3 members, 22 (35.5%) had severe PTSSs. We found that 363 of the 751 respondents who did not have formal education (48.3%) had severe PTSSs. Employed refugees had a lower prevalence of severe PTSSs (113 of 276 [40.9%]) than nonworking participants (430 of 889 [48.4%]). Of the 64 respondents with physical disabilities, 41 (64.1%) had severe PTSSs, while 511 of the 1119 respondents without physical disabilities (45.7%) had severe PTSSs. Also, 281 of 496 respondents who did not receive adequate humanitarian aid during the last 7 days (56.7%) reported severe PTSSs. Moreover, 87 of the 1177 respondents who experienced physical and sexual abuse before displacement (64.0%) reported severe PTSSs.

After categorizing traumatic distress into 2 categories, we calculated the unadjusted PR (Table 2). The PR of mental health symptoms increased with age; with refugees aged 18 to 24 years as a reference, the PR for those aged 25 to 34 years was 1.22 (95% CI, 1.08-1.39), and the PR for those aged 55 years or older was 1.48 (95% CI, 1.28-1.70). Male sex may be a factor associated with severe PTSSs (PR, 1.06; 95% CI, 0.98-1.14). A significant association between receipt of adequate humanitarian aid and PTSSs was observed (PR, 0.75; 95% CI, 0.70-0.81). The results indicate that refugees in Myanmar who experienced both physical and sexual abuse had an increased risk of experiencing severe mental health symptoms (PR, 1.59; 95% CI, 1.43-1.76). Also, results indicated that paid employment in the refugee camps was associated with fewer severe mental health symptoms (PR, 0.91; 95% CI, 0.83-1.01).

**Table 2.** Unadjusted Prevalence Ratios by Sociodemographic, Predisplacement, and Postdisplacement Factors on Traumatic Distress

Characteristic	Participants, No./total No. (%)		Unadjusted prevalence ratio (95% CI)
	Total IES-R score ≥33	Total IES-R score <33	
Age, y			
18-24	150/279 (53.8)	129/279 (46.2)	1 [Reference]
25-34	257/389 (66.1)	132/389 (33.9)	1.22 (1.08-1.39)
35-44	168/209 (80.4)	41/209 (19.6)	1.49 (1.31-1.69)
45-54	150/180 (83.3)	30/180 (16.7)	1.55 (1.36-1.76)
≥55	101/127 (79.5)	26/127 (20.5)	1.48 (1.28-1.70)
Sex			
Female	378/559 (67.6)	181/559 (32.4)	1 [Reference]
Male	448/625 (71.7)	177/625 (28.3)	1.06 (0.98-1.14)
BMI			
Normal	356/509 (69.9)	153/509 (30.1)	1 [Reference]
Overweight or obese	301/431 (69.8)	130/431 (30.2)	0.99 (0.91-1.08)
Underweight	62/96 (64.6)	34/96 (35.4)	0.92(0.78-1.08)
Marital status			
Never married	81/153 (52.9)	72/153 (47.1)	1 [Reference]
Ever married	29/35 (82.9)	6/35 (17.1)	1.56 (1.26-1.93)
Married	715/994 (71.9)	279/994 (28.1)	1.36 (1.16-1.58)
Family members			
≤2	32/62 (51.6)	30/62 (48.4)	1 [Reference]
3-4	218/354 (61.6)	136/354 (38.4)	1.19 (0.92-1.53)
≥5	574/766 (74.9)	192/766 (25.1)	1.45 (1.14-1.85)
Educational level			
1-10 Years of schooling	261/405 (64.4)	144/405 (35.6)	1 [Reference]
Cannot read or write	543/751 (72.3)	208/751 (27.7)	1.12 (1.03-1.22)
Paid employment status in the last month			
Unemployed	631/889 (71.0)	258/889 (29.0)	1 [Reference]
Employed and had earnings	180/276 (65.2)	96/276 (34.8)	0.91 (0.83-1.01)
Physical disabilities			
No	770/1119 (68.8)	349/1119 (31.2)	1 [Reference]
Yes	56/64 (87.5)	8/64 (12.5)	1.27 (1.14-1.41)
Self-reported humanitarian aid (last 7 d)			
Not sufficient	403/496 (81.3)	93/496 (18.8)	1 [Reference]
Sufficient	422/686 (61.5)	264/686 (38.5)	0.75 (0.70-0.81)
Predisplacement abuse			
No	172/293 (58.7)	121/293 (41.3)	1 [Reference]
Physical	202/290 (69.7)	88/290 (30.3)	1.19 (1.05-1.34)
Both physical and sexual	127/136 (93.4)	9/136 (6.6)	1.59 (1.43-1.76)
Verbal or emotional	321/458 (70.1)	137/458 (29.9)	1.19 (1.07-1.34)
Lost immediate family member during predisplacement violence			
No	188/294 (63.9)	106/294 (36.1)	1 [Reference]
Yes	46/48 (95.8)	2/48 (4.2)	1.49 (1.35-1.66)

Abbreviations: BMI, body mass index; IES-R, Impact of Event Scale–Revised.

### Multivariable Analysis: Adjusted PRs

We estimated adjusted prevalence ratios (aPRs) using a multivariable logistic regression model adjusted for potential confounders (Table 3). It appears that sufficient humanitarian aid is associated with reduced risk of symptoms of traumatic distress (aPR, 0.50; 95% CI, 0.38-0.65). The results also suggest that both physical and sexual abuse before displacement were associated with a significant increase in mental health symptoms (aPR, 2.09; 95% CI, 1.41-3.07). After adjustment, results also indicate that paid employment opportunities in refugee camps were assoicated with a reduced risk of developing mental health symptoms (aPR, 0.69; 95% CI, 0.52-0.91).

**Table 3.** Adjusted Prevalence Ratios of Posttraumatic Stress Symptoms After Adjusting for Potential Confounders

Variables	Adjusted prevalence ratio (95% CI)
Age, y	
18-24	1 [Reference]
25-34	1.31 (1.06-1.62)
35-44	1.72 (1.31-2.25)
45-54	1.82 (1.35-2.44)
≥55	1.62 (1.22-2.14)
Sex	
Female	1 [Reference]
Male	1.28 (1.06-1.54)
Marital status	
Never married	1 [Reference]
Ever married	1.64 (1.04-2.59)
Married	1.04 (0.78-1.38)
Family members	
≤2	1 [Reference]
3-4	1.35 (0.94-1.94)
≥5	1.57 (1.08-2.27)
Paid employment status in the last month	
Unemployed	1 [Reference]
Employed and had earnings	0.69 (0.52-0.91)
Self-reported humanitarian aid (last 7 d)	
Not sufficient	1 [Reference]
Sufficient	0.50 (0.38-0.65)
Predisplacement abuse	
No	1 [Reference]
Physical	1.14 (0.92-1.42)
Both physical and sexual	2.09 (1.41-3.07)
Verbal or emotional	0.96 (0.76-1.22)

## Discussion

Nearly 1 million Rohingya people live in the refugee camps of Bangladesh, more than 750 000 of whom have been living there since August 2017. However, compared with 2 years ago, the living conditions in the camps have generally improved, but refugees still live in small, overcrowded temporary shelters in the camps, without sufficient food, clean water, or toilets. Their lives are on hold, and their futures are uncertain. Alongside the traumatic experiences that many Rohingya refugees have experienced, these postmigration factors may contribute to a growing desperation. This study provides a detailed view of the symptoms of traumatic distress encountered by Rohingya refugees in Bangladesh.

Our study indicates that 46.6% of respondents had severe PTSSs and that 23.1% of respondents had moderate PTSSs in the third year after the forced evacuation. In various refugee communities globally, a varying proportion of diagnosed mental health problems has been observed. The prevalence of PTSD in Syrian refugees living in Turkey and Iraq was 83.4% after remaining in the camps for approximately 1 year and 60% after remaining in the camps for approximately 3 years.^32,33^ The prevalence of mental illness was 54% among the war-affected Ugandan population, where two-thirds of that population was displaced for more than 5 years.^34^ Also, 48.8% of Afghan refugees who fled their country nearly 20 years ago and have now lived in Australia for 1 to 5 years have met the criteria for PTSD.^35^ One study of Syrian refugees living in Germany found that only 13% had mental health disorders.^36^ However, a small study conducted in 2017 among camp-based Rohingya refugees who fled to Bangladesh found that 36% of adults had PTSSs and that 89% had symptoms of depression.^21^

There are currently insufficient resources for mental health services in Rohingya refugee settings, and the number of mental health professionals is too low to cover the entire Rohingya population in need.^37^ The high prevalence of PTSSs suggests that a scale-up of mental health care is needed, which could be met by increasing medical workers’ capacity in refugee health facilities to diagnose and treat patients with mental disorders.

In Bangladesh, refugees are not legally entitled to work. The inability to survive without jobs has led many refugees, especially men, to illegally seek paid employment. Many of the Rohingya refugees work in the refugee camps, especially as paid day laborers who help construct roads, prevent landslides, and clear sewage. A few of the refugees have small grocery stores. The paid work allows some Rohingya refugees to supplement their own family’s food rations and provides opportunities to obtain a variety of food. Paid employment offers not just the freedom to buy needed items but also a chance to work with others. Work offers hope that is crucial to healing from mental illness. Our results indicate that those with paid employment are less likely to have symptoms of traumatic distress than unemployed refugees. One meta-analysis found poorer mental health outcomes for refugees with fewer economic opportunities.^38^

Humanitarian aid from the government or NGOs plays an essential role in Rohingya refugees’ lives. The refugees are solely dependent on humanitarian assistance. Our findings show that sufficient humanitarian aid to a family is associated with a lower risk of developing mental health symptoms compared with those who received insufficient assistance. Similar results found that a lack of social support was associated with poorer mental health.^39,40,41^

Before the mass exodus to Bangladesh, sex-based abuse toward refugees was well documented in many reports.^4,5,6^ We found that physical abuse was more frequent among male refugees than female refugees and that both physical and sexual abuse were equally prevalent among male and female refugees. Our findings indicate that, among Rohingya refugees subjected to physical and sexual abuse, the prevalence of severe PTSSs was high. Many studies show that women were found to have a higher incidence of mental health disorders after rape or sexual assault.^38,42^

Approximately half the respondents in our sample were male. The study findings show that symptoms of traumatic distress were more prevalent among male refugees than female refugees. Several studies have shown that male refugees had a higher prevalence of poor mental health.^38,41,42^ A qualitative study among refugees from Afghanistan speculates that the lack of job opportunities plays a crucial role in mental distress among men after years of forced migration.^43^ Rohingya women do not get enough paid jobs in conservative Rohingya society, making it difficult for them to earn a living. Lack of paid employment may be associated with the increase in depression among women. Our research also indicates that refugees with physical disabilities were at a much greater risk of elevated PTSD symptoms than those without disabilities. This finding is consistent with previous studies that found that disability was associated with a high prevalence of mental illness.^44^

In our sample, the risk of traumatic distress increased with increasing age regardless of sex. The findings are consistent with studies conducted among Syrian and Sudanese refugees.^33,45^ Our research also found that refugees who were unable to read or write had a higher prevalence of severe mental health symptoms than those who had schooling. Currently, adult refugees do not have educational opportunities in the refugee camps, and job placement is not based on education. Therefore, among adults in the refugee camps, education has no significance. This finding is comparable to previous research that showed that poor education was correlated with higher rates of PTSD.^46,47^

Also, married respondents were more likely to have PTSSs than those who were never married. This finding is comparable to findings in previous research.^48^ Our study further found a high risk of PTSSs for refugees with family sizes of more than 4 members. The Rohingya population believed that family planning methods clash with their faith.^49^ Thus, married people are more likely to have a large family and be unable to afford enough food or fulfill other needs; this explains why the variables of marital status and family sizes have a positive association with traumatic distress.

### Strengths and Limitations

There are some strengths to our research. First, a local NGO working with Rohingya refugees for more than 3 years helped us collect data from the 8 camps. Our data reduced the information bias by involving interviewers from Rohingya refugee camps. Second, we recruited households through a random sampling technique. Third, to assess symptoms of mental well-being, we collected data on a variety of variables that were not taken into account in current registries or population-scale monitoring efforts.

Several limitations should be taken into account when interpreting our results. First, our research used a scale to measure PTSSs that was not validated for use in the Rohingya community. Second, the method of this short duration of the cross-sectional survey could fail to address the transient nature of the population. Third, we did not assess mental health treatment availability and use in the last 2 years of the postmigration period. This limitation could be significant because accessing or using mental health services may mitigate the prevalence of PTSSs among respondents. Fourth, we did not gather details on the number of assaults experienced by the respondents or the number of many family members who were involved in those events. We had a question concerning the loss of family members during the attack. Unfortunately, with this question, some of the respondents got too emotional to respond, and several respondents misunderstood the question about the concept of immediate family members. We stopped asking these questions later in the study. Similarly, several respondents were worried about their health conditions, but they were not certain about chronic diseases because they did not visit any health facilities. Therefore, many respondents were unable to answer this question. We did not include several postmigration stressors, such as the refugee camp climate, access to health care or education, varieties of food, and limited access to certain services. These variables may have confounding effects on mental health.

## Conclusions

The high prevalence of self-reported mental health symptoms among Rohingya refugees in this study suggests that the trauma of displacement and the violent consequences of military clearance operations are still present. Different support services, such as access to education or training on stress management for their violent memories, may reduce the burden of severe mental health symptoms. Our findings indicate the importance of ensuring that every household receives sufficient humanitarian assistance. Employment opportunities at Rohingya refugee camp settings hold promise as a potential intervention to reduce the burden of mental health symptoms.

## Notes

Supplement. **eTable 1.** Sample Allocation From Kutupalong Rohingya Camp **eAppendix.** Calculation of Response Rate **eReference.** **eTable 2.** Evaluating Multicollinearity by Variance Inflation Factor (VIF) **eFigure.** Mosaic Plot to Understand the Relationship Between Categorical Confounders **eTable 3.** Proportion of Missing Data in Covariates
