# Supplementary material for: Predisplacement Abuse and Postdisplacement Factors Associated With Mental Health Symptoms After Forced Migration Among Rohingya Refugees in Bangladesh
Source: JAMA Netw Open. 2021 Mar 16;4(3):e211801. doi: 10.1001/jamanetworkopen.2021.1801 (PMC7967081; doi:10.1001/jamanetworkopen.2021.1801)
Supplement: Supplement. — eTable 1. Sample Allocation From Kutupalong Rohingya Camp eAppendix. Calculation of Response Rate eReference. eTable 2. Evaluating Multicollinearity by Variance Inflation Factor (VIF) eFigure. Mosaic Plot to Understand the Relationship Between Categorical Confounders eTable 3. Proportion of Missing Data in Covariates [file jamanetwopen-e211801-s001.pdf]

Supplementary Online Content

Hossain A, Baten RBA, Sultana ZZ, et al. Predisplacement abuse and postdisplacement factors associated with mental health symptoms after forced migration among Rohingya refugees in Bangladesh. *JAMA Netw Open.* 2021;4(3):e211801. doi:10.1001/jamanetworkopen.2021.1801

eTable 1. Sample Allocation From Kutupalong Rohingya Camp
eAppendix. Calculation of Response Rate
eReference.
eTable 2. Evaluating Multicollinearity by Variance Inflation Factor (VIF)
eFigure. Mosaic Plot to Understand the Relationship Between Categorical Confounders
eTable 3. Proportion of Missing Data in Covariates

This supplementary material has been provided by the authors to give readers additional information about their work.

© 2021 Hossain A et al. *JAMA Network Open.*

eTable 1. Sample Allocation From Kutupalong Rohingya Camp

**Camps name**	**Households**	**Populaiton**	**Sample size**
Camp 7	5458	44965	145
Camp 4	7191	28263	146
Camp 17	1127	1740	128
Camp 2W	5458	28095	134
Camp 6	5694	27144	160
Camp 5	6153	29789	146
Camp 8E	7730	37500	163
Camp 18		27023	163
		Total	1185

Source of Households and population: Action Contre la Faim Oxfam Save the Children (2018) Rohingya Refugee Response Gender Analysis: Recognizing and responding to gender inequalities Available: https://reliefweb.int/sites/reliefweb.int/files/resources/rr-rohingya-refugee-response-gender-analysis-010818-en.pdf (Accessed on September 15, 2020)

© 2021 Hossain A et al. *JAMA Network Open.*

eAppendix. Calculation of Response Rate

The response rate (RR) is the number of complete interviews with reporting units divided by the number of eligible reporting units in the sample. The following formula was used to calculate the response rate [1].

$$\mathbf{RR1} = \frac{\mathbf{I}}{\mathbf{(I + P) + (R + NC + O) + (UH + UO)}}$$

Where, RR1 = Response rate
I = Complete interview
P = Partial interview
R = Refusal and break-off
NC = Non-contact
O = Other
UH = Unknown if household/occupied HU
UO = Unknown, other

The details of these quantities for our study are given in the following:

I and P=The in-person household survey was conducted in which housing units are sampled from an address-based sampling frame of eight selected camps using systematic sampling technique. We consider less than 50% of all applicable questions answered (with other than a refusal or no answer) equals break-off, 50%-80% equals partial, and more that 80% equals complete. We found complete answered from 1184 participants (i.e., I=1184). We found 14 of the participants did not complete the questionnaire for PTSS (i.e., P=14).

R= Refusals and break-offs consist of cases in which some contact has been made with the housing unit and a responsible household member has declined to do the interview, or an initiated interview results in a terminal break-off. Seventeen of the respondents refused to take part in the interview, but no one took a terminal break-off after initiating an interview (i.e., R=17).

NC= Non-contacts in in-person household surveys consist of three types: a) unable to gain access to the building, b) no one reached at housing unit, and c) respondent away or unavailable. The housings were accessible in the camps and we were able to reach at housing unit during the interview period (i.e., NC=0).

O= Other cases represent instances in which the respondent is/was eligible and did not refuse the interview, but no interview is obtainable because of: a) death; b) the respondent is physically and/or mentally unable to do an interview; c) language problems; and d) miscellaneous other reasons. We did not face any language problem to exclude

© 2021 Hossain A et al. *JAMA Network Open.*

participants. We found 8 physically ill (bed-bound or palliative care) patients from household during the interview (i.e., O=8).

UH= Cases of unknown eligibility and no interview include situations in which it is not known if an eligible housing unit exists and those in which a housing unit exists, but it is unknown whether an eligible respondent is present in the housing unit. It was not a scenario in our situation (i.e., UH=0).

UO= Not eligible cases for in-person household surveys include: a) out-of-sample housing units; b) not-a-housing unit; c) vacant housing units; d) housing units with no eligible respondent; and e) situations in which quotas have been filled. In a systematic sampling technique, we found a total of 57 households without any adult respondents during the interview (UO=57).

Thus, the response rate is, RR=1184/ (1184+14+17+0+8+0+57) = 92.5%

Reference:

[1] The American Association for Public Opinion Research. 2016. *Standard Definitions: Final Dispositions of Case Codes and Outcome Rates for Surveys*. *9th edition*. AAPOR.

© 2021 Hossain A et al. *JAMA Network Open.*

eTable 2. Evaluating Multicollinearity by Variance Inflation Factor (VIF)

```
> glm.full<-glm(PTSD2~factor(Gender)+factor(Age1)+factor(FamilyMember1)+factor(Exploitations1)+
+ factor(Employed_status)+factor(Support),family=binomial,data=data.ptsd.final)

> summ(glm.full,vifs = TRUE)
MODEL INFO:
Observations: 1153 (32 missing obs. deleted)
Dependent Variable: PTSD2
Type: Generalized linear model
  Family: binomial
  Link function: logit

MODEL FIT:
χ²(15) = 203.89, p = 0.00
Pseudo-R² (Cragg-Uhler) = 0.23
Pseudo-R² (McFadden) = 0.14
AIC = 1240.30, BIC = 1321.10

Standard errors: MLE
-------------------------------------------------------------------------------
                                           Est.   S.E.   z val.      p    VIF
----------------------------------------- ------- ------ -------- ------ ------
(Intercept)                               -0.35   0.40    -0.87   0.38
factor(Gender)Male                         0.47   0.16     2.86   0.00   1.36
factor(Age1)25-34                          0.52   0.21     2.44   0.01   1.85
factor(Age1)35-44                          1.24   0.27     4.61   0.00   1.85
factor(Age1)45-54                          1.45   0.28     5.13   0.00   1.85
factor(Age1)55+                            1.05   0.30     3.48   0.00   1.85
factor(MarritalStatus1)Ever-married        1.10   0.56     1.98   0.05   1.77
factor(MarritalStatus1)Married             0.07   0.25     0.26   0.79   1.77
factor(FamilyMember1)3-4                   0.59   0.31     1.86   0.06   1.14
factor(FamilyMember1)4+                    0.96   0.31     3.12   0.00   1.14
factor(Exploitations1)Physical             0.24   0.19     1.21   0.23   1.79
abuse
factor(Exploitations1)Sexual               2.18   0.39     5.61   0.00   1.79
abused
factor(Exploitations1)Verbal              -0.07   0.21    -0.32   0.75   1.79
abuse
factor(Employed_status)Employed           -0.58   0.18    -3.12   0.00   1.32
and have earnings
factor(Disabilities)1                      0.11   0.45     0.24   0.81   1.04
factor(Support)1                          -1.01   0.19    -5.36   0.00   1.55
-------------------------------------------------------------------------------
```

© 2021 Hossain A et al. *JAMA Network Open.*

eFigure. Mosaic Plot to Understand the Relationship Between Categorical Confounders

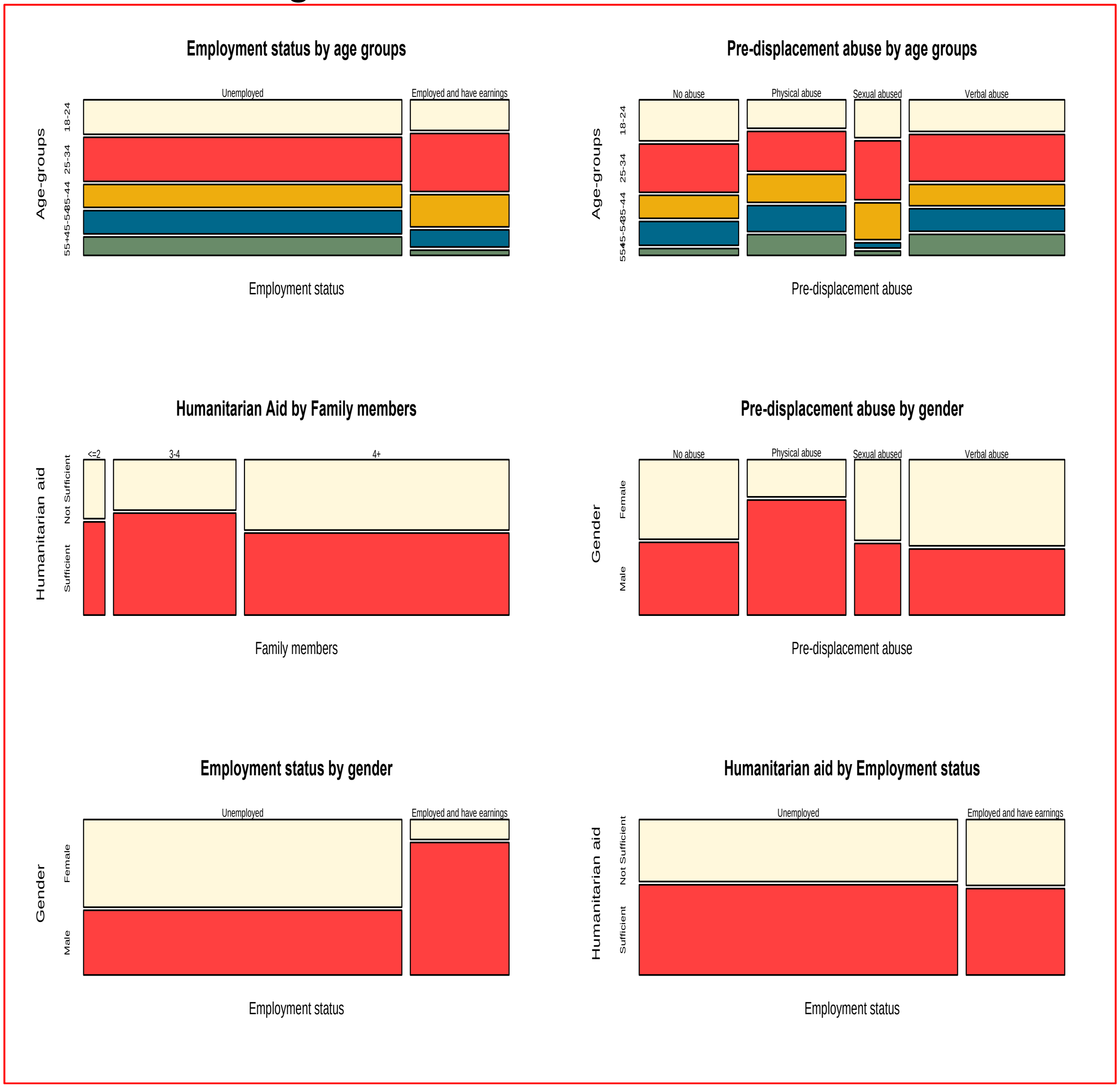

© 2021 Hossain A et al. *JAMA Network Open.*

eTable 3. Proportion of Missing Data in Covariates

**Variable (Available data)**	Missing (%)
**Gender (N= 1184)**	0 (0%)
**Age** (in years) (N= 1184)	0 (0%)
**BMI (N=1036)**	148 (12.5%)
**Marital Status (N=1182)**	2 (0.17%)
**Family size (N=1182)**	2 (0.17%)
**Education (N=1156)**	28 (2.36%)
**Paid-employment status in the last month (N=1165)**	19 (1.60%)
**Physical disability (N=1183)**	1 (0.08%)
**Humanitarian aid for household during the last 7 days (N=1182)**	2 (0.17%)
**Pre-displacement abused (N=1177)**	7 (0.59%)

© 2021 Hossain A et al. *JAMA Network Open.*
